# Follow-up care for cancer survivors: views of the younger adult

**DOI:** 10.1038/sj.bjc.6605213

**Published:** 2009-07-28

**Authors:** K Absolom, C Eiser, G Michel, S J Walters, B W Hancock, R E Coleman, J A Snowden, D M Greenfield

**Affiliations:** 1 Department of Psychology, University of Sheffield, Sheffield, UK; 2 School of Health and Related Research, University of Sheffield, Sheffield, UK; 3 Academic Unit of Clinical Oncology, Weston Park Hospital, Sheffield, UK; 4 Department of Haematology, Royal Hallamshire Hospital, Sheffield Teaching Hospitals Foundation Trust, Sheffield, UK

**Keywords:** cancer survivors, follow-up, models of care, younger adults

## Abstract

**Background:**

Since the launch of the National Cancer Survivorship Initiative, there has been a surge of interest surrounding the value and organisation of long-term follow-up care after cancer treatment. We report the views of 309 adult cancer survivors (aged 18–45 years) on provision of follow-up and preferences for care.

**Methods:**

A total of 207 survivors completed questionnaires before and after routine consultant-led follow-up appointments and 102 were recruited by post. Measures of health status (including late effects, perceived vulnerability to late effects and quality of life), reasons for attending follow-up (clinical and supportive), issues to be discussed at follow-up and preferences for different models of care were assessed.

**Results:**

In all, 59% of the survivors reported experiencing one or more cancer-related health problems. Survivors rated clinical reasons for attending follow-up more highly than supportive reasons (*P*<0.001), although nutritional advice and counselling were considered useful (60 and 47%, respectively). Those still receiving scheduled follow-up appointments did not discuss the range of issues intended with ‘late effects’ and ‘fertility’, which were particularly under-discussed. Hospital rather than GP follow-up was more highly rated.

**Conclusion:**

Survivors value the clinical reassurance currently provided by consultant-led care. However, supportive needs are not systematically addressed. Multi-disciplinary services are recommended to meet supportive needs in addition to clinical care.

Many younger adults aged 18–45 years diagnosed with cancer can expect to live for decades after treatment but face particular problems with regard to fertility and continuing in the work place. Breast cancer is one of the most common malignancies diagnosed in women under the age of 45 years, with a 5-year survival rate now in excess of 80% (Walters *et al*, 2009). In this age range, testicular cancer and Hodgkin's lymphoma also represent a considerable cancer burden, and again 5-year survival rates are very high at ∼98 and 90%, respectively (Nur *et al*, 2008; Walters *et al*, 2009). The good prognosis for this enlarging patient group raises questions with regard to long-term follow-up and delivery of services that are both clinically appropriate and meet individual patient's requirements.

Traditionally, the primary aim of follow-up has been to detect acute problems related to treatment and cancer recurrence, but increasing survival rates have led to a greater understanding of the long-term physical and psychological effects that survivors may experience years after treatment (Stein *et al*, 2008). Termed ‘late effects’, these include infertility, osteoporosis, endocrine dysfunction, fatigue and depression (Kattlove and Winn, 2003; Stein *et al*, 2008). Consequently, follow-up services that identify and treat long-term health implications of cancer have been recommended (Aziz, 2007; Stein *et al*, 2008). To date, there has been little consensus on the value and organisation of follow-up (Montgomery *et al*, 2007). The importance of developing new and improved post-treatment services has been highlighted by The Cancer Reform Strategy (Department of Health, 2007), the subsequent National Cancer Survivorship Initiative and by the NICE Improving Outcomes in Children and Young People with Cancer (National Institute for Health and Clinical Excellence, 2005).

With increasing cancer survival rates, it is essential for long-term care to adapt itself to meet demands. In a paediatric setting, risk-based follow-up has been recommended, whereby patients are stratified to services (primary care based, nurse led or consultant led) depending on their treatment history and likelihood of further health problems (Oeffinger *et al*, 2000; Wallace *et al*, 2001; Geenen *et al*, 2007). A similar approach may also prove to be useful in an adult setting, in which recommendations have been made for the transfer of breast cancer survivors to general practice (National Institute for Health and Clinical Excellence, 2002). Determining patients' views of care or preference for alternative models should be an important consideration before changes to services are implemented. The few studies to date, which have sought to determine patients' opinions, have done so by tumour group (Moore *et al*, 2002; Papagrigoriadis and Heyman, 2003; Dancey *et al*, 2005; Cox *et al*, 2006; Kew *et al*, 2007; Knowles *et al*, 2007), with breast cancer patients evaluated the most (Grunfeld *et al*, 1996; Gulliford *et al*, 1997; Rojas *et al*, 2000; Collins *et al*, 2004; Jiwa *et al*, 2006; Donnelly *et al*, 2007). There has not, however, been a specific focus on how late effects are managed or on survivors' experiences and expectations of follow-up services.

In this study, we first examine survivors' views on their health (current late effects, perceived vulnerability to late effects and quality of life), and second, determine survivors' reasons for attending follow-up and preferences for alternative models of care. Third, survivors attending follow-up appointments were asked about their preferences for further supportive services and the issues they planned to discuss during follow-up appointments.

## Materials and methods

### Eligibility criteria

This was a cohort study of younger adults treated for cancer with curative intent at a regional cancer centre in Sheffield, UK, serving a population of 1.8 million. In the absence of a clear definition of younger adult cancer patients, we specified the age range of eligible participants to be 18–45 years, as younger adults have historically been the explicit focus of our previous research and clinical interest. There is a paucity of evidence examining the efficacy of long-term follow-up in the identification of late effects, particularly in younger adults who are likely to have more complex needs in terms of fertility, duration of survival, employment and family issues. We chose to focus on the main tumours typically diagnosed in younger adults (haematological cancers (lymphoma or acute leukaemia), germ cell or breast cancer). Survivors were required to be a minimum of 5 years from diagnosis without relapse (2 years from diagnosis for the germ cell group). Terminally ill patients and those not fluent in English or unable to provide written informed consent were excluded.

### Procedure

During the study period (from December 2006 to January 2008), eligible survivors were identified from hospital databases and clinic lists. Those receiving follow-up care during this period were contacted by post and invited to participate 1 week before appointments. Consultant-led care was the mode of follow-up received by this group during the course of our study. Completed questionnaires were collected at the clinic before follow-up consultations. Patients attending the clinic were also asked to complete a second questionnaire at home after their appointment. Eligible survivors not currently receiving follow-up care at the specialist centre were contacted by post and invited to participate. Questionnaires completed at home were returned in freepost envelopes.

### Participants

**Figure 1:**
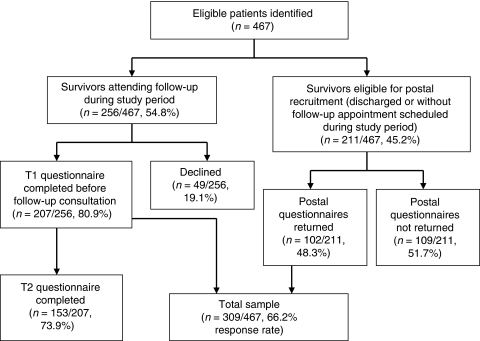
Participant recruitment.

In total, 467 eligible patients were identified (see Figure 1). A total of 256 eligible survivors had follow-up appointments scheduled, of whom 207 (80.9%) completed Time 1 questionnaires and 153 out of 207 (73.9%) returned Time 2 questionnaires. In all, 211 survivors were eligible for postal recruitment only, of whom 102 returned questionnaires (48.3%). In total, 158 survivors declined to participate (breast *n*=37, haematology *n*=39 and germ cell *n*=82). Overall, 309 survivors (response rate=66.2%, breast *n*=75, haematology *n*=131, germ cell *n*=103) participated in this study. The current age of participants (mean=37.9 years) was comparable with those who declined participation (mean=36.9 years, *P*=0.08, difference: −1.02, CI: −2.18 to 0.14).

### Measures

#### Time 1

1. **Demographic information**
2. **Reasons for attending follow-up care:** Survivors rated the importance of follow-up on two scales adapted from Absolom *et al* (2006), measuring (a) clinical care (five items) and (b) supportive care (four items, see Table 4). Items were rated from 1 to 5, with higher scores indicating greater importance.
3. **Supportive services:** Four supportive services identified through a consultation exercise with both clinicians and cancer survivors were listed (support groups, counselling, nutritional advice from a dietician and advice regarding employment/careers). Survivors indicated how helpful they thought each would be on a 5-point scale, with 1 being ‘not at all helpful’ to 5 being ‘very helpful’. Space was also provided for respondents to document other services they thought were helpful.
4. **Issues to be discussed during consultations** (Absolom *et al*, 2006): Ten issues were listed, including current health, medication, fertility, late-effects insurance and an ‘anything else’ option. Survivors were asked whether they wanted to discuss each issue during their next follow-up visit. Total number of issues was computed (0–10).
5. **Current late effects and vulnerability:** A list of 18 possible cancer-related health problems was presented (e.g., infertility, lung damage, fatigue, depression and developed from a measure previously used by Absolom *et al* (2006)). Participants were asked to rate their perceived vulnerability to each problem on a 5-point scale, from ‘very unlikely’ to ‘very likely’. A further alternative response was also provided: ‘I already have this problem’. This scale yields two scores, namely, total number of late effects (0–18) and vulnerability (range 1–5), in which higher scores indicate greater perceived vulnerability.
6. **Health-related quality of life (HRQOL)** The SF-12v2 (Ware *et al*, 2002) is a 12-item measure that can produce two summary scores, namely Physical Component Summary (PCS) and Mental Component Summary (MCS). The PCS and MCS are scaled to have a mean score of 50 and a s.d. of 10, in line with the reference population.

#### Time 2

After clinic appointments, survivors were asked to complete the following measures:

1. **Issues discussed:** The same 11 issues presented at Time 1 were used, and survivors indicated those that they discussed with clinic staff.
2. **Preferences for follow-up care:** To determine the acceptability of different models of follow-up care, survivors were presented with four descriptions of care, namely postal/telephone follow-up, GP led, nurse led and consultant led. Four statements were presented with each description (‘I think this type of care would suit me’; ‘If I had this type of care I would worry that any problems with my health would not be found’; ‘I would be happy with this type of care’; and ‘This type of care would definitely meet my follow-up needs’). Survivors rated how far they agreed or disagreed with each statement on a 5-point scale. Overall sum scores were generated for each form of care (range 4–20), with higher scores denoting a more positive view.

Survivors without scheduled follow-up appointments completed an abridged postal questionnaire consisting of the following measures:

1. demographic information;
2. reasons for attending follow-up care;
3. current late effects and vulnerability;
4. HRQOL (SF-12 v2);
5. preferences for follow-up care.

### Medical information and disease severity

Information on diagnosis, time since completion of treatment and treatment (chemotherapy, radiotherapy or surgery) was obtained for each participant from medical records. Given the heterogeneous nature of the tumour groups, a disease severity scoring system was developed by clinicians from the tumour-specific teams to enable us to describe our population, allowing for comparisons across the different cancers. Individuals were assigned one of three scores: 1=localised (i.e., localised and indolent), 2=intermediate (i.e., generalised indolent/localised aggressive), 3=disseminated (i.e., generalised and aggressive). Cases were allocated by a member of the clinical team. A proportion was rated a second coder, blind to the original decision. Any ambiguous codes were referred to the relevant consultant for a final decision.

### Analysis

Analyses were conducted using SPSS version 11 (SPSS Inc., Chicago, IL, USA). Internal reliabilities of all scales were assessed using Cronbach's alpha. *χ*^2^, *t*-tests and analysis of variance (ANOVA) (with *post hoc* Tukey's tests) were used to compare differences in demographic and clinical variables and questionnaire measures. Age was found to be associated with number of reported late effects and vulnerability scores and was consequently entered as a covariate in univariate analysis, in which the three tumour groups were compared. Multiple regression analysis was used to determine variables associated with the number of reported late effects. The variables entered into the regression were age at study, tumour group (entered as two dummy variables with breast as the reference group) and treatment; surgery (yes/no), chemotherapy (yes/no) and radiotherapy (yes/no). To determine how the sample's HRQOL compared with population data, individual survivor scores for PCS and MCS subscales were matched to the appropriate age- and sex-matched normative SF-12v2 data. Scores were then compared using paired sample *t*-tests. Pearson's correlations were used to identify associations between demographic variables, HRQOL scores, reasons for attending follow-up and number of issues discussed during consultations. The percentage of survivors reporting ‘very important’ for each reason for attending follow-up was used to rank the importance of each item. McNemar's test for paired nominal data was used to compare the percentage of survivors who intended to discuss each issue during their consultation with those who did. A *P*-value of <0.05 represents a significant difference between the two proportions.

## Results

Results are first presented for the entire cohort (assessing current health, reasons for follow-up and follow-up preferences) and second for the sub-sample of survivors who attended scheduled follow-up during the study period and completed items on issues to discuss and supportive services.

Table 1 provides clinical information about the sample, including disease severity scores. Overall, the demographic and clinical characteristics of the sample are in line with those of tumour group populations, with the breast sample being older than the other groups. At the time of the study, the germ cell group was fewer years from treatment than were the other samples. This reflects an earlier routine discharge practice for germ cell survivors than for breast and haematology cancer patients.

### Current health: late effects, perceived vulnerability and HRQOL

Of survivors, 59.2% (183 out of 309, CI: 54–65%) reported one or more late effects as a consequence of their cancer (breast=73.3%, CI: 62–82%; haematology=64.9%, CI: 56–73%; germ cell=41.7%, CI: 33–51%). The mean number of reported late effects for the whole group was 1.6 (Table 2). Survivors with a disease severity score of 3 reported more late effects than did those with scores of 2 or 1 (means=2.0, 1.8 and 1.3, respectively), although differences were not significant (*P*>0.05). After adjusting for age, significantly fewer late effects (Table 2) were reported by germ cell survivors (mean=1.1) than by either breast (mean=1.9) or haematology groups (mean=1.9). The top three most frequently reported late effects by each tumour group are listed in Table 2.

The results of the multivariate regression for total number of reported late effects are shown in Table 3. Older age at the time of study and having received chemotherapy were both associated with more reported late effects.

Vulnerability scores were <3 for all tumour groups (Table 2). After adjusting for age, the germ cell group reported the lowest vulnerability, which was significantly less than that of the haematology group (*P*<0.05) but not of the breast group (*P*=0.30). Women reported higher vulnerability than did men (means=2.7 *vs* 2.5, *P*<0.05, difference: −0.18, CI: −0.34 to −0.03).

In general, survivors compared favourably with age- and sex-matched normative data for SF-12 PCS and MCS. The only exception was the breast group, who reported worse MCS than the normative data (*P*<0.05). More reported late effects were associated with worse PCS (*r*=−0.39, *P*<0.001) and MCS scores (*r*=−0.25, *P*<0.001). Higher perceived vulnerability to late effects was also associated with worse PCS (*r*=−0.27, *P*<0.001) and MCS scores (*r*=−0.32, *P*<0.001).

### Reasons for attending follow-up care

Clinical reasons for follow-up care were more highly rated than were supportive reasons (means=4.6 *vs* 3.6, difference: 1.03, CI: 0.95–1.11; *P*<0.001). The rank order of each of the reasons for follow-up items is shown in Table 4. Checking for cancer recurrence and obtaining reassurance were the most highly rated reasons and getting advice with regard to everyday matters was the least. Women scored significantly higher than did men for both scales (clinical means=4.7 *vs* 4.6, difference: −0.10, CI: −0.19 to −0.01; *P*<0.05; supportive means=3.7 *vs* 3.5, difference: −0.22, CI: −0.41 to −0.02; *P*<0.05). There were no significant differences between the three disease severity groups (clinical means: level 1=4.6, level 2=4.6, level 3=4.6, *P*=0.95; supportive means: level 1=3.5, level 2=3.6, level 3=3.5, *P*=0.43). The scores for participants with follow-up appointments during the study period were not significantly different than for those without (clinical means 4.6 *vs* 4.5, difference: 0.09, CI: −0.01–0.19; supportive means=3.6 *vs* 3.5, difference 0.03, CI: −0.18–0.24). The breast sample rated supportive reasons significantly more highly than did the germ cell group (3.8 *vs* 3.5, *P*<0.05); there were no other differences between the tumour groups. Higher supportive scores were associated with higher vulnerability (*r*=0.24, *P*<0.001).

### Preferences for alternative models of follow-up

Regardless of tumour group or disease severity, consultant-led care was preferred over nurse-led, telephone follow-up and GP-based care (see Table 2). There were also no associations between follow-up preferences and disease severity, number of reported late effects, vulnerability or HRQOL (data not shown).

### Supportive services and issues discussed during consultations

Nutritional advice from a dietician was the most highly rated of the support services, with 59.9% (124 out of 207, CI: 53–66%) of survivors reporting that they would find this useful. The next most popular service was counselling (46.9%, 97 out of 207; CI: 40–54%), followed by support groups (45.9%, 95 out of 207; CI: 39–53%) and employment/careers advice (28.5%, 59 out of 207, CI: 23–35%). Additional services of value that survivors identified included advice on exercise, holistic therapies, physiotherapy, as well as medical tests and information (including advice on fertility, late effects and reducing the risk of cancer recurrence).

The total number of issues that the survivors intended to discuss at Time 1 and Time 2 did not differ across tumour groups (Time 1 mean scores: breast=3.5, haematology=3.4, germ cell=3.6, *P*=0.80; Time 2 mean scores: breast=2.3, haematology=2.6, germ cell=2.3, *P*=0.48). Survivors attending follow-up appointments who returned Time 2 questionnaires (*N*=153) reported discussing significantly fewer issues during consultation than they had intended (means=3.4 *vs* 2.4, difference: 1.01, CI: 0.64–1.4; *P*<0.001). Table 5 shows the percentage of survivors intending to discuss each issue before their consultation compared with what they reported discussing afterwards. There were clear discrepancies between Time 1 and Time 2 percentages for late effects, health behaviours, fertility and insurance.

The number of issues that the survivors intended discussing was associated with higher ratings for supportive (*r*=0.48, *P*<0.001) and clinical reasons for follow-up (*r*=0.26, *P*<0.001), as well as with higher feelings of vulnerability (*r*=0.23, *P*<0.01). In comparison, the number of issues they reported actually discussing during consultations was associated with more reported late effects (*r*=0.21, *P*<0.05) and worse PCS (*r*=−0.20, *P*<0.05).

## Discussion

Although these young adult cancer survivors reported comparable HRQOL with age- and sex-matched normative data, ∼60% experienced cancer-related problems. On an average, the sample was 8 years from treatment, but expectations with regard to follow-up were still focussed on checking for cancer recurrence and on obtaining reassurance. In concordance with this finding, hospital-based follow-up was more highly rated than was the prospect of transfer to general practice, although many survivors were in favour of access to supportive services, including advice from a dietician and counselling.

The breast cancer survivors were the only group to report significantly worse HRQOL than normative data. This was for mental rather than for physical health, and supports findings from previous research suggesting younger breast cancer survivors fare worse psychologically than do their older counterparts (Ganz *et al*, 2003). Indeed, a considerable proportion of breast cancer survivors in this study reported problems with mood, as well as with weight gain and lymphoedema. Commonly reported problems in the rest of the sample included infertility, chronic fatigue and gonadal dysfunction. Although the germ cell group reported fewer late effects than did both the breast and haematology survivors, regression analysis indicated that older age at the time of study and having received chemotherapy were the two variables significantly associated with more reported late effects. These findings reflect the wide range of health issues effecting survivors and the complexity of providing follow-up services, which meet both the physical and psychological needs of individual patients in the long term.

Across all tumour groups, checking for cancer recurrence and getting reassurance were rated as the most important reasons for attending follow-up care. Supportive issues were consistently ranked lower, a finding that is in line with clinicians' views of the purpose of continued care (Greenfield *et al*, 2009). After the end of treatment, recurrence is a major fear for many patients; hence, this finding is not surprising. However, attending follow-up may provide a false reassurance, as evidence suggests recurrences can often be detected between scheduled follow-up appointments (Grunfeld *et al*, 1996). In addition, our patient sample was typically many years from the end of treatment (mean 8.1 years) and the risk of recurrence is therefore low.

Regardless of tumour group, number of reported late effects or disease severity, preferences for follow-up showed a marked bias towards hospital-based services over telephone or GP-based care. This is in contrast to recent recommendations, including those for breast cancer patients, proposing the transfer of survivors to primary care (National Institute for Health and Clinical Excellence, 2002). Given the fact that our sample only had experience of consultant-led care and that the other proposed models were hypothetical, it is understandable that many would show allegiance to specialist services in which they are both familiar and have confidence. The introduction of alternative models of follow-up, such as nurse-led or GP-based services, will have to contend with the ethos (created by both patients and staff) that consultant care is the optimal option, despite the fact that former models may be better equipped to meet supportive care needs. In a systematic review of breast cancer follow-up, Montgomery *et al* (2007) report that patients followed up by breast cancer nurses report higher satisfaction than do those receiving care from doctors, and that QOL is similar between the two groups. The recent draft NICE breast cancer guidelines (National Institute for Health and Clinical Excellence, 2009) call for greater patient choice in defining follow-up services. However, for an informed choice, patients may require a better understanding of the purpose of follow-up and the roles that different health-care professionals can have in assisting with clinical and supportive problems. Importantly, the evidence suggesting that cancer recurrence is typically detected by patients between follow-up appointments needs to be relayed to patients and supported with advice on the recognition of symptoms that require clinical assessment and possible investigation.

In spite of the emphasis placed on cancer recurrence, survivors expressed a wish to discuss a range of issues and concerns during follow-up consultations. Besides general matters with regard to current health and medication, many wanted to discuss late effects and fertility, and a considerable percentage hoped for advice with regard to health behaviours. Despite this, most survivors reported the discussion of significantly fewer issues than they had indicated before consultations. Most strikingly, 36% of survivors reported discussing late effects, although 78% had hoped to. The total number of issues that the survivors intended to discuss was associated with personal views of follow-up and perceived vulnerability to late effects. In contrast, the number of issues they reported discussing was associated with more medical variables (number of late effects and physical health). This suggests that a medical agenda and time limitations may be the most influential in the content of follow-up consultations. Further qualitative research may be necessary to examine survivors' opinions on what governs the content of consultations and why certain topics are not discussed. Patients may be reluctant to raise the discussion of emotional and supportive issues in consultations, fearing they are wasting the doctor's time. In recognition of this, a recent study describes the benefits of asking head and neck cancer patients to complete a ‘concerns inventory’, the findings of which are informed to the consultants (Rogers *et al*, 2009). This approach may be one way of matching the content of consultations to patient preferences more accurately.

Our results indicate that health behaviours were not frequently discussed during consultations, despite the patient's desire to do so. Thus, health professionals are missing an opportunity to promote healthy living. Although there is a growing body of literature highlighting the role clinicians should undertake in counselling survivors on the adoption of healthy lifestyles (Bellizzi *et al*, 2005; Demark-Wahnefried *et al*, 2005; Sabatino *et al*, 2007), there is no definitive guidance in this area. As with many follow-up services, the survivors in our study were mainly seen in clinics alongside patients on active treatment. Under the typical time pressures many clinicians face, greater attention is understandably focussed on acutely ill patients. Many doctors may not see it as their role to deliver general lifestyle advice to survivors. However, a significant proportion of the current sample wanted to discuss health behaviours and ∼60% said nutritional advice would be a useful addition to current follow-up care. Perhaps a different forum is required to provide the supportive services survivors seem to want, alongside clinical reassurance with regard to their health. Specialist nurses and GPs may be more suitably positioned and experienced to either provide this type of care or effectively sign-post to appropriate services and agencies (Greenfield *et al*, 2009). Future follow-up care needs to address how best to integrate these multi-disciplinary services.

This study involved a sizeable sample of younger adult cancer survivors with particular age-associated needs from representative tumour groups. It is the first to specifically explore views of follow-up services in this population. Our study had a number of limitations. First, survivors were recruited from a single cancer centre and may not be representative of patients from other centres. Second, we have relied on survivors' self-reports of current late effects and topics discussed during consultations without medical data or doctor's reports to verify results. Third, the issues of follow-up that we surveyed were generic rather than tumour-specific issues. For example, there are recognised issues pertinent to each tumour group, such as monitoring endocrine therapy for breast cancer survivors, that require a more detailed tumour-specific approach but were not addressed in this survey. Fourth, we acknowledge the fact that the generalisability of our findings is limited to the age range we studied, which represents only 10% of the entire cancer population (Horner *et al*, 2008). However, the excellent survival rates for many cancers affecting younger adults, coupled with their complex requirements in terms of fertility, employment and family issues, present unique challenges for the development of long-term follow-up care. Fifth, we recruited survivors several years from the end of treatment, whose needs and perceptions may differ from those who have more recently completed treatment. Finally, we had limited clinical information on survivors who declined to participate in this study and hence are unable to establish how representative our non-responders were compared with the study participants. Although challenging, future research should aim at determining the views and needs of survivors who routinely miss appointments and those who become lost to follow-up, as they may be a particularly vulnerable group.

In concordance with users involved with the think-tank exercise to launch the National Cancer Survivorship Initiative ‘Living with and beyond cancer’ (March 2008), our results highlight the wide range of follow-up needs occurring after cancer. Future research may take into account the possible benefits of alternative or complementary follow-up models. Consideration should be given to both the economic impact of different models, as well the training needs of health-care professionals delivering care. Although the survivors involved in this study were satisfied with consultant-led follow-up with its clinical focus, we recommend that future services take a multi-disciplinary approach to encompass both clinical and supportive needs across primary, secondary and tertiary services.

## Figures and Tables

**Table 1:** Study sample

	**Breast *N*=75**	**Haematology *N*=131**	**Germ cell *N*=103**	**Total *N*=309**
*Demographics*				
Age (s.d.)	41.2 (3.1)	37.9 (5.6)	35.7 (6.6)	37.9 (5.8)
Years since end of treatment (s.d.)	6.2 (2.4)	11.8 (5.1)	4.7 (3.0)	8.1 (5.1)
Male % (*n*/*N*)	0 (0/75)	50.4 (66/131)	92.2 (95/103)	52.1 (161/309)
				
*Treatment % (n/N)*				
Surgery	100.0 (75/75)	41.2 (54/131)	98.1 (101/103)	74.4 (230/309)
Chemotherapy	90.7 (68/75)	88.5 (116/131)	50.5 (52/103)	76.4 (236/309)
Radiotherapy	81.3 (61/75)	72.5 (95/131)	34.0 (35/103)	61.8 (191/309)
				
*Disease severity % (n/N)*				
1 (localised disease)	60.0 (45/75)	18.3 (24/131)	64.1 (66/103)	43.7 (135/309)
2 (intermediate disease)	40.0 (30/75)	55.7 (73/131)	26.2 (27/103)	42.1 (130/309)
3 (disseminated disease)	0 (0/75)	26.0) (34/131)	9.7 (10/103)	14.2 (44/309)

**Table 2:** Late effects, vulnerability, health-related QOL (SF-12v2) scores and preference for follow-up

		**Breast (*N*=75)**	**Haematology (*N*=131)**	**Germ cell (*N*=103)**	**Total (*N*=309)**
Late effects^a^ *range 0–18*	Total mean (CI)	1.9 (1.5–2.4)	1.9 (1.6–2.2)	1.1 (0.7–1.5)	1.6 (1.4–1.9)
Most common late effects % (*n*/*N*)	First	Weight gain	Infertility	Testicular/ ovarian damage	
		32.0 (24/75)	28.2 (37/131)	26.2 (27/103)	
	Second	Lymphoedema	Thyroid damage	Infertility	
		30.7 (23/75)	21.4 (28/131)	12.6 (13/103)	
	Third	Mood swings	Chronic fatigue	Hearing loss	
		29.3 (22/75)	17.6 (23/131)	10.7 (11/103)	
					
Vulnerability to late effects^b^ *range 1–5*	Mean (CI)	2.6 (2.4–2.7)	2.7 (2.6–2.8)	2.4 (2.3–2.5)	2.6 (2.5–2.6)
Health-related QOL^c^	Physical component summary (CI)	52.8 (50.9–54.7)	52.1 (50.6–53.7)	53.7 (52.0–55.4)	52.8 (51.8–53.8)
	Mental component summary (CI)	45.3 (42.8–47.8)	48.7 (47.1–50.3)	51.0 (49.3–52.7)	48.6 (47.5–49.7)
		**Breast (*N*=71)**	**Haematology (*N*=102)**	**Germ cell (*N*=82)**	**Total (*N*=255)**
Follow-up preferences mean (CI)	Consultant led	16.4 (15.8–17.0)	17.2 (16.7–17.7)	17.0 (16.4–17.6)	16.9 (16.6–17.2)
*Range 4–20*	Nurse led	12.9 (12.0–13.9)	13.7 (13.0–14.5)	13.4 (12.6–14.2)	13.4 (12.9–13.9)
	Telephone/ questionnaire	10.0 (8.9–11.0)	10.2 (9.3–11.1)	11.3 (10.4–12.2)	10.5 (10.0–11.0)
	GP based	9.6 (8.5–10.6)	9.4 (8.5–10.2)	9.8 (8.9–10.8)	9.6 (9.0–10.1)

CI=confidence interval; GP=general practitioner; MCS=Mental Component Summary; PCS=Physical Component Summary; QOL=quality of life.

a Means adjusted for age, germ cell <breast (*P*<0.05) and haematology (*P*=0.01)

b Means adjusted for age, germ cell <haematology (*P*<0.05).

c The PCS and MCS are scaled to have a mean score of 50 and s.d. 10 in line with the reference population.

**Table 3:** Multivariate regression analysis showing variables associated with total number of reported late effects

	**Unstandardised B**	***P*-value**	**95% confidence intervals**
Age at study	0.055	0.008	0.02–0.10
Breast *vs* haematology	0.145	0.681	−0.55–0.84
Breast *vs* germ cell	−0.504	0.193	−1.26–0.26
Surgery	0.240	0.478	−0.43–0.91
Radiotherapy	−0.012	0.964	−0.55–0.52
Chemotherapy	0.667	0.037	0.04–1.29

**Table 4:** Rank order of survivors' ratings of reasons for attending follow-up

**Ranks**	**Item**	**% Survivors reporting very important**	**Scale (C=Clinical, S=Supportive)**
1	Check for cancer recurrence	90.3 (279/309)	C
2	Get reassurance about health	74.1 (229/309)	C
3	Get the best medical care	68.3 (221/309)	C
4	Help staff learn more about late effects of cancer	50.2 (155/309)	C
5	Get information about late effects	42.1 (130/309)	C
6	Talk to staff who understand what I have been through	23.6 (73/309)	S
	Get advice about how to keep healthy	23.0 (71/309)	S
7	Receive psychological support	16.8 (52/309)	S
8	Get advice about everyday things, such as insurance	10.7 (33/309)	S

**Table 5:** Percentage of survivors (*N*=153) intending to discuss each issue before follow-up consultation and those they report discussing

	**T1 Intend to discuss % (*n*)**	**T2 Report discussing % (*n*)**	***P*-value**
Current health	86.3 (132)	86.9 (133)	0.728
Late effects	77.8 (119)	35.9 (55)	<0.001
Health behaviours	47.7 (73)	32.7 (50)	0.007
Fertility	32.0 (49)	15.0 (23)	<0.001
Medication	31.4 (48)	30.1 (46)	1.000
Insurance	23.5 (36)	2.0 (3)	<0.001
Work/education	15.7 (24)	13.1 (20)	0.597
Sexual problems	12.4 (19)	5.2 (8)	0.019
Contraception	11.8 (18)	3.9 (6)	0.001
